# Limitations of malaria reactive case detection in an area of low and unstable transmission on the Myanmar–Thailand border

**DOI:** 10.1186/s12936-016-1631-9

**Published:** 2016-11-25

**Authors:** Daniel M. Parker, Jordi Landier, Lorenz von Seidlein, Arjen Dondorp, Lisa White, Borimas Hanboonkunupakarn, Richard J. Maude, François H. Nosten

**Affiliations:** 1 Shoklo Malaria Research Unit, Mahidol-Oxford Tropical Medicine Research Unit, Faculty of Tropical Medicine, Mahidol University, Mae Sot, Tak Thailand; 2 Mahidol-Oxford Tropical Medicine Research Unit, Faculty of Tropical Medicine, Mahidol University, Bangkok, Thailand; 3 Nuffield Department of Medicine, Centre for Tropical Medicine and Global Health, University of Oxford, Oxford, UK; 4 Faculty of Tropical Medicine, Mahidol University, Bangkok, Thailand; 5 Harvard T.H. Chan School of Public Health, Harvard University, Boston, USA

**Keywords:** *Plasmodium*, Reactive case detection, Disease ecology, Geographic information science, Simulation

## Abstract

**Background:**

Reactive case detection is an approach that has been proposed as a tool for malaria elimination in low-transmission settings. It is an intuitively justified approach based on the concept of space–time clustering of malaria cases. When an index malaria clinical case is detected, it triggers reactive screening and treatment in the index house and neighbouring houses. However, the efficacy of this approach at varying screening radii and malaria prevalence remains ill defined.

**Methods:**

Data were obtained from a detailed demographic and geographic surveillance study in four villages on the Myanmar–Thailand border. Clinical cases were recorded at village malaria clinics and were linked back to patients’ residencies. These data were used to simulate the efficacy of reactive case detection for clinical cases using rapid diagnostic tests (RDT). Simulations took clinical cases in a given month and tabulated the number of cases that would have been detected in the following month at varying screening radii around the index houses. Simulations were run independently for both falciparum and vivax malaria. Each simulation of a reactive case detection effort was run in comparison with a strategy using random selection of houses for screening.

**Results:**

In approximately half of the screenings for falciparum and 10% for vivax it would have been impossible to detect any malaria cases regardless of the screening strategy because the screening would have occurred during times when there were no cases. When geographically linked cases were present in the simulation, reactive case detection would have only been successful at detecting most malaria cases using larger screening radii (150-m radius and above). At this screening radius and above, reactive case detection does not perform better than random screening of an equal number of houses in the village. Screening within very small radii detects only a very small proportion of cases, but despite this low performance is better than random screening with the same sample size.

**Conclusions:**

The results of these simulations indicate that reactive case detection for clinical cases using RDTs has limited ability in halting transmission in regions of low and unstable transmission. This is linked to high spatial heterogeneity of cases, acquisition of malaria infections outside the village, as well missing asymptomatic infections. When cases are few and sporadic, reactive case detection would result in major time and budgetary losses.

**Electronic supplementary material:**

The online version of this article (doi:10.1186/s12936-016-1631-9) contains supplementary material, which is available to authorized users.

## Background

In regions with decreasing malaria burden, the distribution of malaria becomes increasingly patchy and heterogeneous across both landscape and population [1, 2]. Given increased efforts towards elimination and evolving epidemiological characteristics, novel approaches at identifying and treating malaria cases may be necessary for disrupting transmission [3].

Reactive case detection (RCD) uses information about passively detected malaria index cases in order to target potential secondary cases [4]. When a symptomatic malaria patient is diagnosed at a health facility, health care workers screen household members, neighbours and other related community members for malaria. This form of case detection is an intuitive approach in that if cases of malaria cluster in space and time, then it may be efficient to target those clusters in order to disrupt transmission [5, 6]. Such an approach is not considered feasible or efficient in high-transmission settings, but may be appropriate for interrupting transmission in a patchy environment [7, 8].

Several studies in a variety of settings (e.g., Zambia, Peru, Thailand) have found strong clustering of malaria within households [9–12]. From this finding it seems logical that reactively screening or presumptively treating individuals who share households with index cases will result in the treatment of cases that could have otherwise been missed. Several projects have explored screening of neighbouring households at varying radii around an index house. For example, studies in Sri Lanka [13] and Swaziland [14, 15] led to a suggested 1-km radius around index households for RCD. Modelling work from Zanzibar suggested that RCD would need to target approximately 100 neighbouring households around each identified case in order to halt transmission [16]. Data from Zambia have suggested clustering of cases within households and up to 50 m around index households [12]. Modelling work from Zambia has suggested that more than three-quarters of cases may be detected through reactively screening in a 500-m radius around index households [17].

In most nations of the Greater Mekong Subregion, RCD is listed as a component of national strategic plans for malaria [18]. However, it is most commonly used as part of research projects with external funding rather than by government officials. If the approach is effective it may be important to consider its inclusion in routine malaria control and elimination activities. Conversely, if the approach fails to halt transmission it could divert important financial and labour resources that could better be directed to other approaches. The relative effectiveness of the RCD approach lies in a balance between the proportion of cases it can detect and treat in a timely manner and the feasibility of the approach, especially with regard to resources (funding, labour, social concerns) needed in order to reach that proportion [12].

Here, empirical clinical malaria case data were used to simulate RCD strategies using varying screening radii, time intervals between activities, and settlement characteristics in order to assess the effectiveness and efficacy of RCD to disrupt transmission along the Myanmar–Thailand border.

## Methods

### Data

**Fig. 1:**
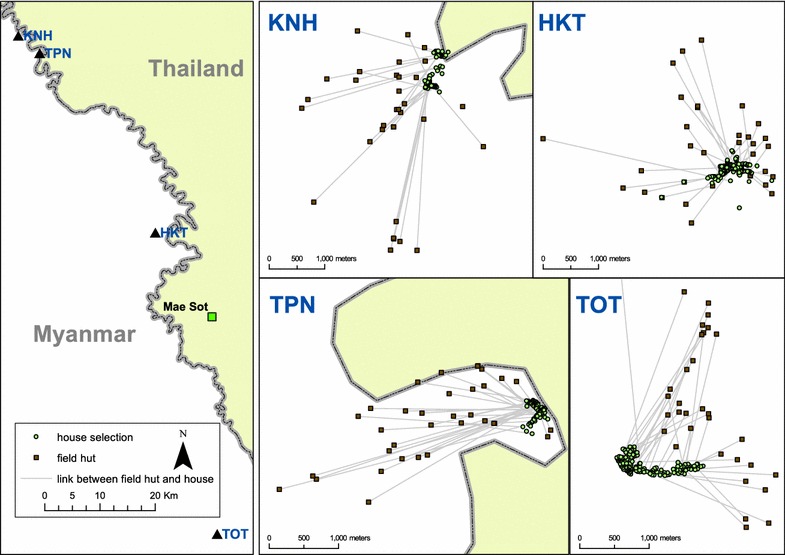
Location of villages along the Myanmar–Thailand border. Individual maps indicate both the locations of houses (*green points*) and farm huts (*brown points*) which are linked to their respective houses using lines

The data were obtained from four villages in Kayin State, Myanmar, located within 10 km of the Myanmar–Thailand border (Fig. 1). The distance from the northernmost village to the southernmost village is approximately 100 km; two villages are within 6 km of each other. The study period began in May 2013 and ended in June 2015. The villages were part of a longitudinal study on asymptomatic malaria carriage and targeted chemo-elimination.

A baseline census and geographic survey were completed at the beginning of the study period, including geographic references (latitude and longitude) for each house in the villages. The populations are primarily composed of agriculturalists and each of the study villages is surrounded by farms, the primary crop being paddy rice. During peak labour times in the farming season many villagers move into farm huts. Geographic surveys therefore targeted these farming huts as well, marking their locations with global positioning system (GPS) units and linking them to houses within the villages. Identification numbers were assigned to houses and participants so individual participants could be linked back to their respective houses.

Community-based malaria clinics were established in each village at the beginning of the study period. Patients who presented at the clinic (the only source of diagnosis and treatment in the villages) and were part of the village study had their identification numbers recorded by malaria clinic staff. Patients were tested using a rapid diagnostic test (RDT) (SD Bioline) and those who were diagnosed with malaria infections were treated with dihydroartemisinin-piperaquine (DP) for *Plasmodium falciparum* and chloroquine for *Plasmodium vivax* infections. Radical cure (using primaquine) was not provided for vivax infections because of a high prevalence of glucose-6-phosphate dehydrogenase deficiency in the population [19]. These empirical clinical case data were used to simulate different RCD scenarios.

The cases included in this research are symptomatic and RDT positive and a limitation to this work is the absence of information about asymptomatic cases. In order to assess the sensitivity of the results to this limitation, data were used from mass blood screenings that were completed in the study villages every 3 months. The screenings included the use of RDTs regardless of symptoms and also recorded the current existence of fever as well as a history of fever within the previous 2 days (self-reported).

### Settlement patterns

Distance matrices were calculated for the locations between houses in each of the villages and for the villages including the farm huts. Summary statistics were calculated from the distance matrices as descriptions of the settlement patterns of villages. All calculations were done using R software [20].

### Temporal patterns in clinical malaria

Repeated clinical cases of both falciparum and vivax within houses in the study villages were analysed. Individuals who presented at the village-based malaria clinic were linked to their house using the demographic surveillance system. Household members in houses in which a clinical case (index case) occurred during the study period were selected as a cohort. The time until a new case within the same house (secondary case) occurred was analysed, and then expanded to a 50-m radius around the home of the case and increasing radii by 50-m intervals up to a maximum of 500 m. The median time between consecutive falciparum cases within a house (roughly 1 month) was used as the base time step (the time interval used for the timing of screening) for the main simulations. The time step of one month indicates that a screening will occur in the month following the month in which index cases occurred. The sensitivity of the analytic results was tested by also using a 1-week time unit.

### Simulations

Using the space–time clinical case data collected from village clinics, four different RCD approaches (three main simulations and one comparison) were simulated. The three main simulations included: a 1-month time step, a 1-month time step but with the settlement expanded to include farming huts, and a 1-week time step. As a comparison, a simulation was also run using the 1-month time step and selecting houses at random rather than using spatial targeting around index houses. The simulations looked for the potential cases that would have been detected given the location of index cases that would have triggered reactive screening. Simplistic time steps of 1 month were used, with an assumption that screening would be constant during the entire month and that all cases that were originally detected through the community-based clinic would also have been detected through reactive screening.

**Fig. 2:**
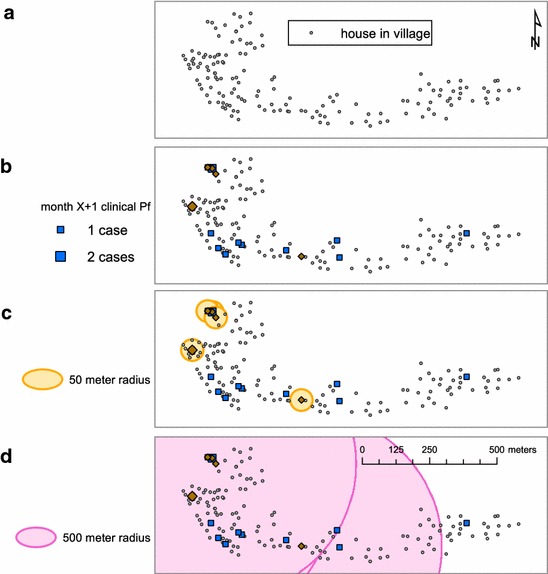
Maps indicating simulation steps. Index houses are selected in step **a**. During the next time step (**b**) secondary houses that overlap index houses are selected and the number of clinical cases in secondary houses is totalled. The process is then repeated by looking at secondary houses within buffers around the index houses beginning with a 50-m radius (**c**) and moving up to 500-m radius (**d**) around index houses

Briefly, houses within which an individual clinical case occurred during a given time step (time ‘X’) were considered ‘index’ houses. These index houses were used as the basis for reactive screening in the following time step interval (time ‘X + 1’), with continuous screening occurring during that time interval. When a house was identified as an index house in time X (e.g., the first month), the number of cases (symptomatic RDT-positive cases from the community-based malaria clinic) in time ‘X + 1 (e.g., the subsequent month)’ were tabulated. Subsequent iterations tabulated cases in time X + 1 within varying radii around index houses, beginning with a 50-m radius and toggling up to houses which fell within 500-m radius of index houses (Fig. 2).

Simulations were run on each village in isolation and the final results were pooled. The simulations were run separately for clinical vivax and falciparum cases. Households with multiple farms were assigned to the farm house that was furthest away from the village. Not every house within a village had an associated farm hut (24% of all houses were linked to a farm hut). Therefore, the simulations that included farm huts were a combination of settlements, including both village houses and farm huts.

Simulation results include: the proportion of all clinical cases that would have been detected in a given time step and the corresponding proportion of all houses that would have been screened, given the location of index houses and varying radii in the previous time step. The spatial simulations were run using the Python programming language [21] and ArcGIS 10.2 [22].

### Ethics approval

This research was approved by the Oxford Tropical Research Ethics Committee (1015-13, dated 29 Apr 2013), the Tak Border Karen Advisory Board and village committees for each study village.

## Results

### Summary spatial distributions of houses within villages

The median distance between any two houses within the same village ranged from 153 to 400 m and the maximum distance was 3141 m (Table 1). Half of all houses were within at least 400 m of all other houses in the same village, meaning that a screening radius of larger than 400 m would usually result in screening most (median 95%) houses in a village.

**Table 1:** Summary distance statistics (in m) for houses in study villages

	Min	Q1	Median	Mean	Q3	Max	Houses
TOT	4.40	170.70	345.90	428.70	692.90	1202.00	154
KNH	5.27	129.50	400.10	428.30	589.50	1813.00	87
TPN	3.02	86.90	153.40	174.50	234.40	755.00	75
HKT	4.13	154.00	290.00	681.10	806.10	3141.00	184

The inclusion of farm huts created disproportionate expansions across the four study villages (Table 2). For example, the maximum distance between settlements (defined here has ‘farm huts’ and ‘village houses’) in Village TOT expanded over 8½ times, whereas there was very little change in the maximum distance between settlements in Village HKT [which had the largest maximum distance between houses (Table 1)]. In most of the study villages the median distance between settlements doubled (Table 2) in comparison to the median distance between houses alone (Table 1). This would correspond to a rough doubling of village geographic space during the agricultural, rainy season.

**Table 2:** Summary distance statistics (in m) for houses and farm huts in study villages

	Min	Q1	Median	Mean	Q3	Max	Settlements
TOT	4.40	319.00	844.30	1584.00	2112.00	10,360.00	214
KNH	5.27	281.50	613.30	962.80	1364.00	4080.00	114
TPN	3.02	157.20	454.60	875.20	1468.00	3854.00	108
HKT	4.13	195.10	436.60	861.60	1529.00	5381.00	214

### Time until next clinical case, by radius and malaria species

Out of a total of 56 falciparum infections, 20 occurred in houses in which there had previously been a falciparum infection (Table 3). In houses with repeat falciparum infections, the median time until the next infection was 39 days (Table 3). Out of a total of 137 vivax infections, 75 occurred in houses in which there had previously been a vivax case. In houses with repeat vivax infections the median time until next vivax infection was 68 days (Table 4). The median time until the next infection was inversely proportional to the radii around a house. For example, the median time until a secondary falciparum infection within 250-m radius of an index house was 14 days and the median time until a secondary falciparum infection within 500-m radius was 9.5 days (Table 3). In vivax index houses the median time until the next vivax infection within 250-m radius was 9 days and for a 500-m radius was 7 days (Table 4). As the search radius widens, it is more likely that there will be a secondary case detected within the search radius and that the time until a secondary case is detected will be reduced.

**Table 3:** Time (in days) until next *Plasmodium falciparum* infection by radius (in metre)

Radius	Min	Q1	Median	Mean	Q3	Max	Secondary infections
House	2.0	17.0	39.0	91.6	139.0	307.0	20
50	1.0	14.5	31.0	70.1	107.0	307.0	34
100	1.0	9.8	27.5	50.3	44.0	281.0	44
150	1.0	9.8	22.5	64.0	50.5	584.0	48
200	1.0	6.0	16.0	51.2	34.0	584.0	49
250	1.0	5.0	14.0	46.4	32.0	584.0	49
300	1.0	5.0	14.0	46.8	32.0	584.0	49
350	1.0	5.0	11.0	37.6	25.0	584.0	49
400	1.0	4.0	10.0	34.5	22.0	584.0	49
450	1.0	4.0	9.5	33.7	21.8	584.0	50
500	1.0	4.0	9.5	33.7	21.8	584.0	50

**Table 4:** Time (in days) until next *Plasmodium vivax* infection by radius (in metre)

Radius	Min	Q1	Median	Mean	Q3	Max	Secondary infections
House	3.0	26.0	68.0	118.5	184.5	604.0	75
50	1.0	18.5	43.0	85.6	112.5	451.0	115
100	1.0	10.0	22.0	47.4	66.0	293.0	129
150	1.0	6.0	15.5	36.1	33.8	446.0	128
200	1.0	5.0	11.0	26.5	24.3	446.0	128
250	1.0	4.0	9.0	28.8	26.5	405.0	128
300	1.0	3.0	8.5	21.1	23.0	405.0	130
350	1.0	3.0	7.0	18.0	19.0	405.0	130
400	1.0	3.0	7.0	16.9	18.0	405.0	130
450	1.0	3.0	7.0	16.7	18.0	405.0	130
500	1.0	3.0	7.0	16.7	18.0	405.0	130

## Simulation results

### Month time step, village only

With a 24-month study period and four study villages, there were a total possible 96 village/month combinations. In many of these village/month combinations no malaria cases occurred. When a month with no malaria cases occurred followed by a month in which a reactive screening was triggered, there was no possibility to find malaria cases.

**Fig. 3:**
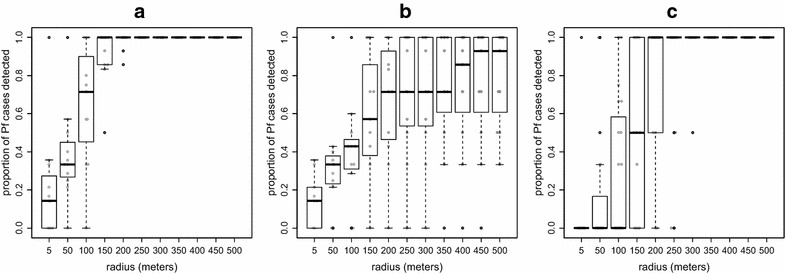
Simulation results for proportion of all *Plasmodium falciparum* (Pf) detected by radius. **a** Using month time steps, **b** using month time steps but including farm huts, **c** using week time steps instead of month time steps. At the month time step (**a**, **b**), 54% of the screenings would have occurred during months in which no case occurred, and at the week time step (**c**) 41% of the screenings would have occurred during weeks in which no cases occurred. The results depicted here are for the remaining 46% (for **a**, **b**) and 59% (**c**) of screenings during which it was possible to detect cases. The 5-m radius indicates screening only within the index house

Out of the 96 village/month combinations, there were 24 village/months during which there was at least one falciparum case and this would have resulted in a screening during the following month. In 13 (56%) of these screenings no case would have been detected, regardless of the screening radius used, because there was no case during that month. In the remaining 11 screenings, approximately 70% (median: 71%; min–max: 0–100%) of symptomatic *P. falciparum* cases occurring within 1 month of the index case would have been detected within a radius of 100 m around index houses. Almost all cases (median: 100%; min–max: 50–100%) would have been detected at 150-m radius (Fig. 3a). Using a 150-m radius around falciparum index houses would have resulted in screening around 75% of all houses (median: 75%; min–max: 18–100%) within the respective study villages.

**Fig. 4:**
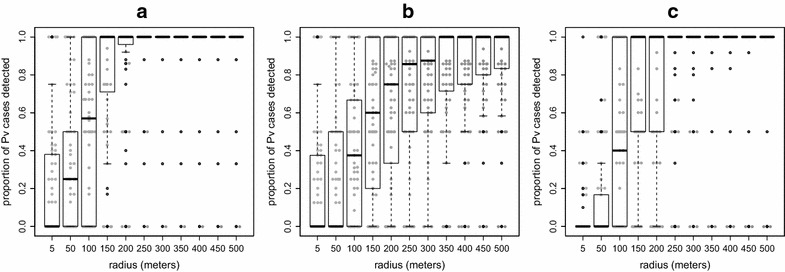
Simulation results for proportion of all *Plasmodium vivax* (Pv) detected by radius. **a** Using month time steps, **b** using month time steps but including farm huts, **c** using week time steps instead of month time steps. 5-m radius indicates screening only within the index house. At the month time step (**a**, **b**), 10% of the screenings would have occurred during months in which no case occurred and at the week time step (**c**) 45% of the screenings would have occurred during weeks in which no cases occurred. The results depicted here are for the remaining 90% (for **a**, **b**) and 55% (**c**) of screenings during which it was possible to detect cases

There were 72 village/month combinations during which at least one vivax case occurred, resulting in a screening in the following month. In seven of the following months (10%) there were no cases to be detected. In the screening months during which cases occurred (the remaining 90%) the majority of *P. vivax* cases (median: 100%; min–max: 0–100%) would have been detected using a 150-m radius around index houses (Fig. 4a) and would have resulted in screening approximately 79% of all houses (median: 79%; min–max: 8–100%) within the respective study villages. The range of outcomes at 150-m radius was wider for vivax cases in comparison to falciparum cases.

### Month time steps including farm huts

As in the previous simulations using month time steps, 54 and 10% of all falciparum and vivax screening events, respectively, would have occurred during months with no clinical cases, resulting in no possible detections regardless of screening radius size. During months in which cases could be detected, the inclusion of farm huts increased the radius size necessary to detect high proportions of cases. Less than 80% of falciparum cases (median: 71%; min–max: 0–100%) would have been detected at 250-m radius (Fig. 3b). At 400-m radius over 80% of all cases (median: 86%; min–max: 0–100%) would have been detected and roughly 75% of all houses (median: 75%; min–max: 2–86%) would have been screened. Over 85% of vivax cases (median: 86%; min–max: 0–100%) would have been detected using a 250-m screening radius (Fig. 4b). This same radius would have resulted in screening roughly 70% of all houses (median: 69%; min–max: 2–86%) in the respective villages.

### Week time step

There were 49 village/weeks (out of 416 possible) during which at least one falciparum case occurred and would have triggered a screening in the following week. In 20 (41%) of those subsequent screening weeks there were no secondary falciparum cases to be detected. During weeks in which there were cases to detect, only half (median: 50%; min–max: 0–100%) of all *P. falciparum* cases would have been detected at 150-m radius and most (median: 100%; min–max: 0–100%) would have been detected at 200-m radius (Fig. 3c). This would have resulted in screening 53% (median: 53%; min–max: 18–96%) and 73% (median: 735; min–max: 29–100%) of all village houses at 150- and 200-m radius, respectively.

There were 170 village/weeks during which vivax cases occurred and in 77 (45%) of the following weeks there were no vivax cases to be detected. Among the remaining 93 weeks in which there were vivax cases to detect, most cases (median: 100%; min–max: 50–100%) would have been detected at a radius of 150 m (Fig. 4c). At the same radius approximately 55% of all houses (median: 55%; min–max: 1–100%) would have been screened. Out of the 170 screenings this would have triggered, 77 would have occurred during weeks when no vivax cases occurred.

### Comparison of randomly selected houses and targeted screening

Spatial targeting of houses was more effective at smaller radii. In the first round of simulations (using the month time step and houses in the village) screening only in index houses in the subsequent month would have resulted in detecting 14% (median: 14%; min–max: 0–100%) of all clinical falciparum cases and almost no vivax cases (median: 0%; min–max: 0–100%). Conversely, randomly selecting the same number of houses for screening would have resulted in detecting almost none of the falciparum cases (median: 0%; min–max: 0–17%) and none of the vivax cases (median: 0%; min–max: 0–33%) (Additional files 1, 2). Spatial targeting continued to outperform random screening until the screening radius reached approximately 200 m. At 200-m radius almost all falciparum (median: 100%; min–max: 50–100%) and all vivax (median: 100%; min–max: 0–100%) would have been detected by both.

### Asymptomatic cases

From the cross-sectional screening data, there were a total of 28 individuals who were RDT-positive for falciparum malaria and 222 who were positive for vivax during the study period. Out of these individuals, eight (29%) of the falciparum-positive were febrile and 37 (17%) of the vivax-positive were febrile, meaning that approximately 71% of falciparum and 83% of vivax positive individuals were asymptomatic during the screenings.

## Discussion

Infectious diseases sometimes cluster in space and time and if it is possible to predict the location of clusters it may also be possible to halt transmission through early diagnosis and treatment using an RCD approach [1, 6]. RCD is now a part of the national strategic plans of most Greater Mekong Subregion countries, though the approach (e.g., how RCD is triggered, the screening radius used) varies greatly by location [18].

The results here indicate that in houses with consecutive malaria cases, recurrent falciparum cases occur more quickly in comparison to vivax cases. Using a 1-month time step would have led to detecting a higher proportion of falciparum cases within index houses when compared to vivax cases. This may indicate within-house clustering of falciparum malaria. Also, many vivax infections in these populations are the result of relapse and such cases are less likely to exhibit strong spatial clustering. However, the results of using screening radii from 150 m and above are similar between *P. falciparum* and *P. vivax* cases. At this radius the majority of cases would have been detected while screening roughly three-quarters of the houses for both falciparum and vivax malaria. When the time step was decreased to a week it resulted in the same result for vivax (detected most cases while screening about three-quarters of all houses at 150-m radius), but would have only detected about half of all falciparum cases.

In this low-transmission context, 50% of screenings would have occurred in months during which there would have been no falciparum cases to find (median time between index and secondary cases within houses: 39 days; within a 150-m radius: 21 days, Table 3). When there were cases to be found, there was no optimum radius that maximized the number of cases found compared to number of houses treated: house-level screening led to a minimal number of houses screened but only identified 14% of secondary falciparum cases. On the other hand, above 150-m radius, a large proportion of cases was found by screening a large proportion of the village. At such a large proportion of village screened, targeted screening around an index case only had a marginal gain compared to randomly screening the same proportion of village households. These results are consistent with recent findings from Pailin Province, Cambodia where few cases were detected through reactively screening index houses and neighbours [23].

The relative success achieved in detecting larger proportions of cases at larger screening radii must also be weighed against the costs incurred through using larger radii. Screening requires labour and medical supplies (e.g., testing and diagnosis materials). If cases occur within a single village at different times it means there will be overlap in screening. A house that was tested in a previous screening could fall within the screening radius of a subsequent index case, requiring repeated screening. Not only does this require the use of large amounts of medical supplies and labour, it would also mean that participants may need to give blood frequently.

Unless transmission is relatively high, most people who are screened will test negative using standard RDTs. If people are asked to frequently take the time to give their blood and be tested for malaria and they are only rarely malaria positive, it could lead to participation fatigue and even refusal. The same situation can lead to fatigue amongst community-based health workers who may see their efforts as relatively unfruitful (given low numbers of cases that would normally be detected) and even unwanted from the community in a scenario of increasing participant avoidance and refusal. The results could be detrimental not only for malaria efforts but also efforts towards control or elimination of other diseases.

The spatial distribution of malaria cases in groups of houses can arise through complicated transmission patterns. For example, most falciparum cases are likely to be new infections whereas many vivax infections are likely to be relapses. Many vivax cases are therefore the result of exposure that happened in the distant past and clustering is unlikely. Exposure to infectious mosquitoes may occur in different locations. If transmission occurs within the village, clusters of houses with cases can be expected around mosquito breeding sites [6]. In contrast, if transmission occurs outside of the village, e.g., in outlying farms, then patterns within the village are related to shared exposures outside of the village. For example, clustering within households may be the result of shared exposure among household members outside of the village. If most transmission occurs outside of the village there may be very little clustering of cases between houses and spatial targeting within the village may not be effective or efficient. These data indicate spatial clustering within and across houses at very brief points in time, with dispersed spatial patterns in between these brief points in time.

The proportion of symptomatic cases detected increases with the screening radius size. At radii in which most cases are being detected (for example, 150-m radius) most of the inhabitants of the afflicted communities would also be tested. If there are multiple index houses, and if those houses are not clustered in one portion of the village, a screening radius of 150 m is sufficient to cover most of the village. At larger screening radii the location of the index house(s) is increasingly irrelevant as the majority of the village will be included. At 200-m radius, targeted screening is no better than randomly selecting the same proportion of houses.

Furthermore, population densities of these villages and many others in the region change seasonally. During times of the year when farming work is at its peak, usually during the rainy and malarious season, many villagers spend long hours in farm fields and spend nights in their farm huts. The population density of the village is dispersed at these times in an anisotropic fashion. Farms are not evenly distributed around villages but rather follow the contours of the landscape (Fig. 1), along fertile soil patches and in places that are deemed appropriate (e.g., legally and socio-culturally) for farming. Cases can occasionally occur within groups of farm huts. The screening radius necessary to detect high proportions of clinical cases increases heterogeneously and is dependent on the settlement patterns of farm huts and the locations of clinical cases. While these measurements of villager destinations outside of the village are most likely an underestimate, they already show that RCD is limited when accounting for movements outside of the village.

There are several limitations to this work. As with RCD, the clinical cases in this study were detected through the use of RDTs and symptomatic patients that presented at community-based clinics. RDTs miss low-parasitaemia cases, which may be important for ongoing malaria transmission. The simulations also miss those asymptomatic cases with high enough parasitaemia to be RDT positive. Mass blood screenings in the same study villages revealed 71% of falciparum and 82% of vivax RDT-positive villagers to be without fever. These findings are likely to be a conservative estimate of the proportion of symptomatic RDT-positive villagers. Many villagers with symptomatic malaria presented at the clinics and were no longer ill at the time of screening (cases on which the simulations were based). Furthermore, the only symptom considered during screenings was fever. Some of the RDT-positive villagers that are considered asymptomatic here are likely to have exhibited symptoms (e.g., headache, nausea) other than fever but are included in the proportion asymptomatic reported above.

Another limitation is that this approach assumes that screening is constant across the time interval (e.g., month). This is an oversimplification used for the simulation. There were two main reasons for choosing the month time step. Foremost was that preliminary analysis indicated repeat cases of falciparum malaria within a single house tend to occur at monthly intervals (Table 3). This roughly corresponds to the time that would be necessary for a symptomatic primary infection to lead to a symptomatic secondary infection (including incubation periods within both mosquito and human). At the community level, cases occur in uneven time intervals with several cases occurring in a single day followed by days or weeks of no cases, even during months in which there were cases to be detected. If an RCD were triggered by each symptomatic case, it would more frequently result in screenings during days or weeks during which there were no cases to detect. Aggregating the simulation cases into month intervals therefore creates a scenario in which there are fewer times steps with no cases to detect.

Alternatively, RCD including detection of asymptomatic cases may be more effective with a shorter time step as treatment of these cases may prevent them going on to develop symptoms and also further reduce ongoing transmission. However, the efficacy of such a strategy would be limited by the poor sensitivity of RDTs for asymptomatic infections. A strategy that relied on detection of asymptomatic carriers would require a more sensitive diagnostic, ideally PCR to be optimally effective. This would be more expensive, would result in a longer turnaround time between testing and results and be much less practical for use in the field. While there have been major improvements in the sensitivity and specificity of RDTs over the last several years, they still miss many low parasitaemia cases [11, 24, 25]. Novel RDTs are in development, which will allow the detection of much lower parasite densities than currently available RDTs. The use of more sensitive RDTs is likely to increase the impact of RCD.

The locations of farm huts in these simulations are an underestimate of villager time spent outside of the home village, as only villagers with farm huts in near proximity to the village were identified (within several km). Results here should take into account the fact that villagers work in locations much further than these farm huts, meaning that small screening radii would likely be even more inefficient than shown through these simulations.

The simulations also could not control for the impact of treating cases in one time step on the occurrence of cases beyond the next time step. Finding and treating current cases should decrease subsequent cases, but the magnitude of this effect is difficult to quantify. However, all patients with clinical malaria in this study were diagnosed and treated, meaning that this effect should be built into the data that fed the simulations. RCD will only halt transmission if diagnosis and treatment are able to be offered quickly. For now this means that RCD is dependent on RDTs that miss many sub-microscopic malaria infections.

These data suggest that the efficacy for reducing malaria transmission through an RCD approach based on RDTs to detect symptomatic cases is likely to be low in this setting. More intensive approaches, including detection and treatment of asymptomatic cases, may be more efficacious but require far more resources and are challenging to implement, especially in resource-poor settings. Other major public health approaches rely on the distribution of bed nets and community-based health clinics. Bed nets have shown limited effectiveness in the region, most likely because of the diversity of mosquito vectors and the exophilic behaviour of several species [26, 27]. Based on current knowledge, the most appropriate strategy for malaria elimination in most communities in this region is a strong community-based primary health clinic (village health post) in which villagers will receive appropriate early diagnosis and treatment for malaria upon the appearance of symptoms [28].

## Conclusions

While attractive in theory, the screen-and-treat approach for RCD of malaria has major limitations. Several previous studies which have evaluated the screen-and-treat approach for malaria control and elimination have invariably failed to detect a significant impact [26–31]. While some studies have indicated that the RCD approach can lead to detecting more cases than normal passive case detection [32, 33], it has not yet shown an ability to halt transmission and in some settings appears to be ineffective [34]. This analysis, based on clinical case data and simulated RCD for clinical cases using RDTs, indicates that the RCD approach in this setting is not effective. In conclusion, spatial targeting approaches require occurrence of spatiotemporal clustering of cases within villages, or in this scenario also in farm huts. Using large screening radii will result in finding most cases, but at potentially much higher financial cost, requiring much effort on behalf of public health workers, and expecting that community members will be willing and available to participate in frequent screenings even in situations where few or no cases will be detected. Ultimately the RCD approach in this setting is limited because of these economic and social costs and because of current limitations in quick malaria diagnosis.

## Additional files

**Additional file 1.** Proportion of falciparum cases that would have been detected using random selection of the same proportion of houses selected by a given radius around index houses.

**Additional file 2.** Proportion of vivax cases that would have been detected using random selection of the same proportion of houses selected by a given radius around index houses.

## Abbreviations

RCD: reactive case detection
RDT: rapid diagnostic test
DP: dihydroartemisinin-piperaquine
GPS: global positioning system
