# Supplementary figures and images for: Limitations of malaria reactive case detection in an area of low and unstable transmission on the Myanmar–Thailand border
Source: Malar J. 2016 Nov 25;15:571. doi: 10.1186/s12936-016-1631-9 (PMC5124267; doi:10.1186/s12936-016-1631-9)

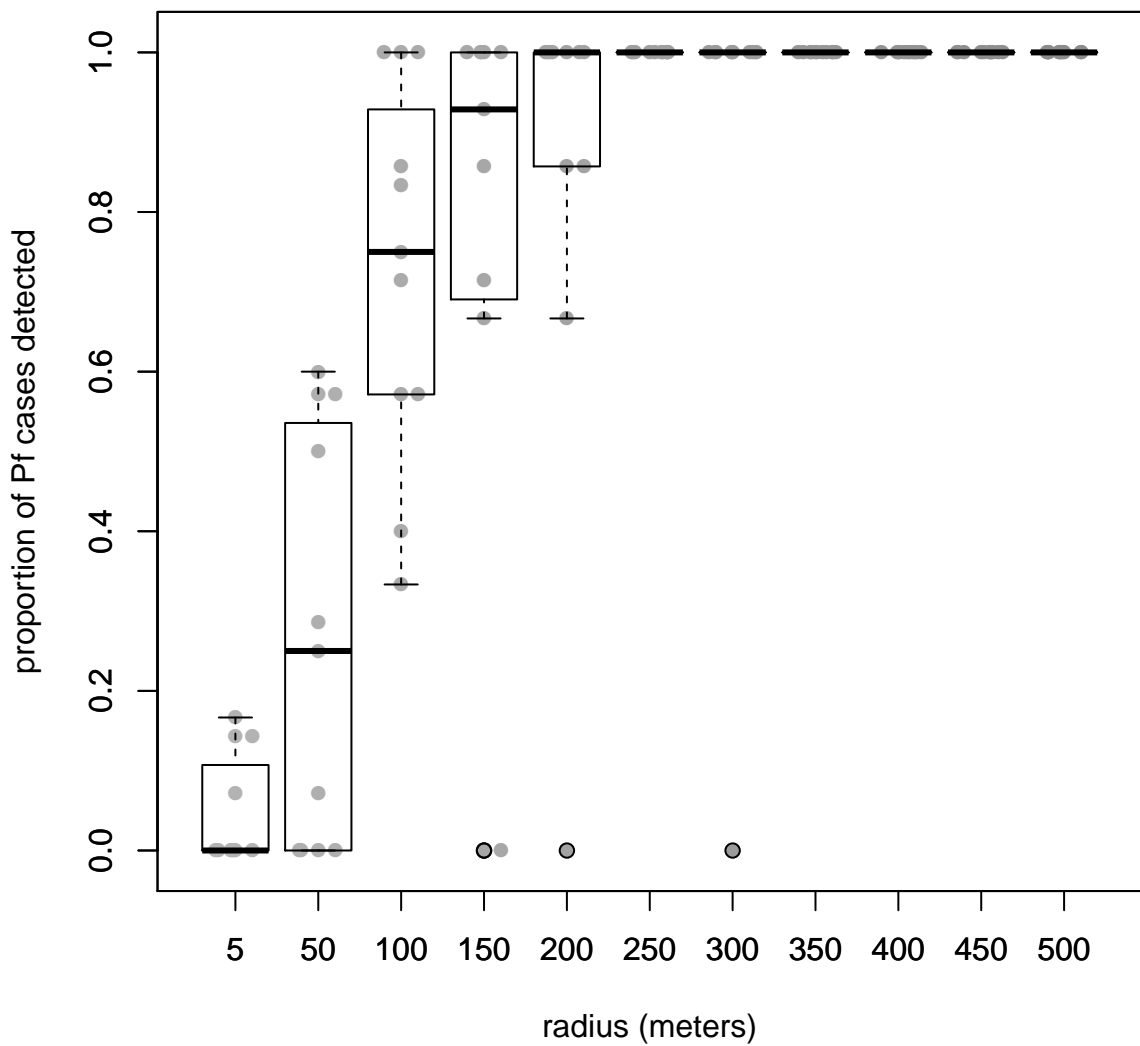

Supplement: Supplementary file 1 — Additional file 1. Proportion of falciparum cases that would have been detected using random selection of the same proportion of houses selected by a given radius around index houses. [file 12936_2016_1631_MOESM1_ESM.pdf]

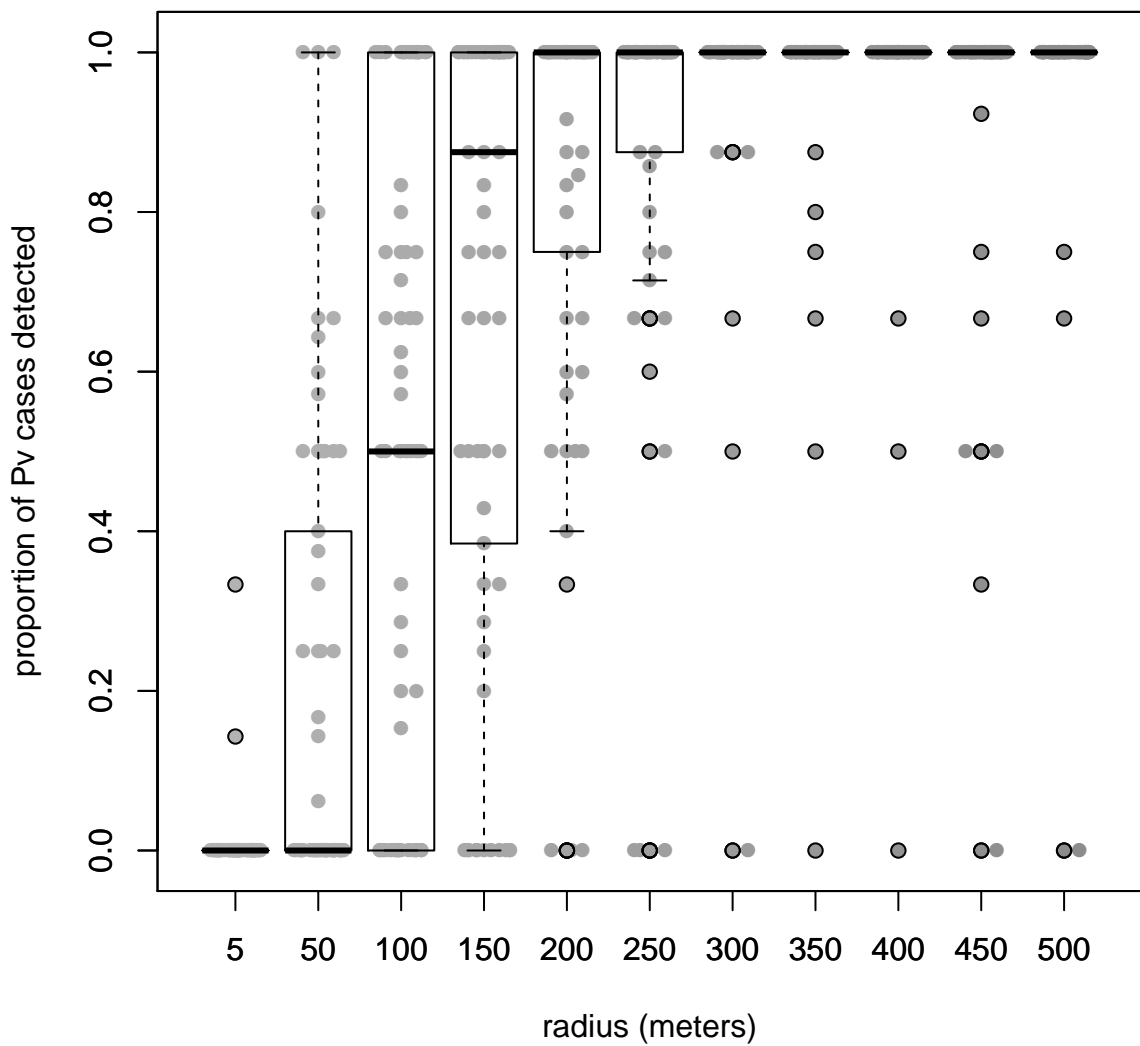

Supplement: Supplementary file 2 — Additional file 2. Proportion of vivax cases that would have been detected using random selection of the same proportion of houses selected by a given radius around index houses. [file 12936_2016_1631_MOESM2_ESM.pdf]
